# Neuroanatomical Correlates of Emotion-Related Impulsivity

**DOI:** 10.1016/j.biopsych.2022.07.018

**Published:** 2022-08-04

**Authors:** Matthew V. Elliott, Serajh A.S. Esmail, Kevin S. Weiner, Sheri L. Johnson

**Affiliations:** Department of Psychology (MVE, SASE, KSW, SLJ) and Helen Wills Neuroscience Institute (KSW), University of California at Berkeley, Berkeley, California.

## Abstract

**BACKGROUND:**

Emotion-related impulsivity (ERI) refers to chronically poor self-control during periods of strong emotion. ERI robustly predicts psychiatric disorders and related problems, yet its neuroanatomical correlates are largely unknown. We tested whether local brain morphometry in targeted brain regions that integrate emotion and control could explain ERI severity.

**METHODS:**

One hundred twenty-two adults (ages 18–55 years) with internalizing or externalizing psychopathology completed a structural magnetic resonance imaging (MRI) scan, the Three-Factor Impulsivity Index, and the Structured Clinical Interview for DSM-5. The Three-Factor Impulsivity Index measures two types of ERI and a third type of impulsivity not linked to emotion. Cortical reconstruction yielded cortical thickness and local gyrification measurements. We evaluated whether morphometry in the orbitofrontal cortex (OFC), insula, amygdala, and nucleus accumbens was associated with ERI severity. Hypotheses and analyses were preregistered.

**RESULTS:**

Lower cortical gyrification in the right lateral OFC was associated with high ERI severity in a full, preregistered model. Separate examinations of local gyrification and cortical thickness also showed a positive association between gyrification in the left lateral OFC and ERI. An integrated measure of hemispheric imbalance in lateral OFC gyrification (right < left) correlated with ERI severity. These findings were specific to ERI and did not appear with non–emotion-related impulsivity.

**CONCLUSIONS:**

Local gyrification in the lateral OFC is associated with ERI severity. The current findings fit with existing theories of OFC function, strengthen the connections between the transdiagnostic literature in psychiatry and neuroscience, and may guide future treatment development.

For centuries, religion, philosophy, and science have debated the etiology of impulsivity. All humans behave impulsively, but some more than others. Impulsivity is not a unitary construct, but rather includes several separable dimensions (1,2) that are products of gene-by-environment interactions (3) and show trait-like stability in people (4,5). One phenotype, emotion-related impulsivity (ERI), which is defined by frequent loss of control during strong emotion states (6), is a robust, transdiagnostic predictor of internalizing disorders (2,7,8) (e.g., depression), externalizing disorders (2,7,9) (e.g., substance abuse), aggression (10–12), and suicidality (13,14), with stronger effects than other forms of impulsivity, such as difficulty planning ahead, staying on task, and sensation seeking. These effects are as potent as those for other well-established psychiatric risk factors, such as neuroticism (2,15–17). Because ERI contributes to the development of psychopathology across mental health disorders (18–20), there is a profound need for progress in understanding its neuroanatomical correlates. However, nearly 2 dozen functional and anatomical magnetic resonance imaging (MRI) studies have failed to discover a consistent neurobiological profile of ERI (21).

We consider 4 factors likely contributing to the inconsistent cross-study results regarding the neuroanatomical profile of ERI. First, most studies have taken an exploratory, whole-brain approach, which may diminish statistical power and increase the risk of false positive findings. Second, most studies have been limited by small sample sizes (*n* < 50), with only 3 studies including more than 100 participants (22–24). In the largest study of more than 10,000 scans from the ABCD (Adolescent Brain Cognitive Development) Study sample (22), significant findings emerged in brain regions that are theorized to integrate emotion and cognitive control, for example, the orbitofrontal cortex (OFC) (25) and insula (26). The effect sizes, however, were very small (Δ*R*^2^s ≤ 0.0033) and were diluted by a constellation of results in regions without ties to previous findings or theory (e.g., middle temporal gyrus, para-hippocampal gyrus, and cuneus). Thus, this study, which may have been affected by the developmental stage of the sample and the suboptimal psychometric properties of the impulsivity scale used (22,27), did not provide clarity regarding the discrepancies previously observed in the literature. Third, structural MRI studies that did not include participants with mental health concerns may not represent those with severe ERI; null neurocognitive results have been more common in studies of nonclinical than clinical samples (21,28). When clinical samples were included in neuroanatomical studies, researchers recruited participants from single diagnostic categories (e.g., schizophrenia or substance use disorder) (11,29–31). As ERI is elevated transdiagnostically, the use of transdiagnostic samples might yield more robust findings and guard against third variable confounds specific to any one diagnosis. Fourth, although researchers have primarily focused on volume-based anatomical features, surface-based features, such as cortical thickness (CT) and local gyrification index (LGI), have enhanced our understanding of other brain-behavior links (32–34). For example, LGI, which has not been used to study ERI, is a critical marker of cortical development (35,36), interacts with genetic (37) and environmental (38) factors, and is related to executive functioning and reasoning abilities (39,40). Beyond the need to address inconsistencies in findings, recent work has identified a second form of ERI, Pervasive Influence of Feelings (PIF), with differentiable and robust impacts on mental health outcomes (7,41), but neuroimaging studies have only considered Feelings Trigger Action (FTA) (i.e., urgency) and not PIF.

This study addressed each of these critical factors. To do so, we recruited a transdiagnostic sample with a broad range of internalizing and externalizing psychopathologies. We conducted preregistered, theory-driven analyses focused on specific brain regions and promising surface-based brain measures. To our knowledge, this study is the first to investigate PIF in a neuroimaging study. We hypothesized that the strongest morphological correlates of ERI would be in the OFC. We hypothesized that OFC morphology would not relate to a non–emotion-related facet of impulsivity used as a control comparison.

## METHODS AND MATERIALS

### Participants

One hundred thirty adults of ages 18–55 years (mean = 28) participated in a parent study approved by the UC Berkeley Committee for the Protection of Human Subjects. Individuals in the general community experiencing impairment from a wide range of internalizing and externalizing mental health symptoms [Sheehan Disability Scale (42) >5 in at least 1 life setting] were recruited through flyers, online advertising, and referrals from clinicians. Individuals with a history of bipolar disorder or primary psychosis or with current alcohol or substance use disorders [as assessed by the Structured Clinical Interview for DSM-5 (43)] were excluded. Other exclusionary criteria included the daily use of marijuana or sedating medications (including antipsychotics), lifetime head trauma resulting in a loss of consciousness for 5 or more minutes, diminished cognitive abilities [Orientation Memory Concentration Test score < 7 of 12 (44)], MRI safety contraindications (e.g., ferrous metal in body, pregnancy, seizure disorders), neurological disorders, or inability to complete cognitive measures independently owing to intellectual or language problems.

Participants who met these criteria were invited to the university to complete diagnostic, behavioral, and neuroimaging sessions. All participants completed informed consent procedures and urine toxicology screens to exclude those with recent consumption of drugs of abuse before scanning. Of the 130 participants who completed structural MRI scans, 8 were removed after visual inspection revealed artifacts in their reconstructed cortical surfaces (*n* = 122). Table 1 describes the sample demographic and clinical characteristics and descriptive statistics for the impulsivity measure.

### Measures and MRI Acquisition

#### Three-Factor Impulsivity Index.

Trait impulsivity was measured using the well-validated Three-Factor Impulsivity Index (3,7), which is composed of the following three-factor analytically based subscales: FTA, PIF, and Lack of Follow Through (LFT). The first two indices measure impulsive responses to emotion, while the third is composed of items reflecting impulsivity without reference to emotion. More specifically, FTA captures a pattern of overt, regrettable action or speech in response to emotion and is composed of items from the Negative Urgency scale (1), the Positive Urgency Measure (45), and the Reflexive Reactions to Feelings scale (3). PIF captures patterns of unconstrained cognitive and motivational responses and is composed of items from the Generalization (46), Sadness Paralysis (3), and Emotions Color Worldview (3) scales. LFT reflects impulsivity without reference to emotion and is composed of items from the Lack of Perseverance (1) and Distractibility (3) scales. All 3 factors show strong internal consistency (3,10,13,47). The 2 scales that reference emotion, FTA and PIF, consistently relate more strongly to measures of psychopathology than does LFT (7,13,41). Therefore, our hypotheses focus on the ERI scales, with LFT used as a control comparison. Univariate impulsivity distributions are depicted in Figure S1. Intercorrelations of the 3 factors (*r*) ranged from 0.23 to 0.38, comparable to other published datasets (Table S1) (3,48).

#### Structured Clinical Interview for DSM-5.

The Structured Clinical Interview for DSM-5 is a semistructured interview that is commonly used to assess psychopathology (43). Participants completed the Structured Clinical Interview for DSM-5 in person or by Zoom interview during the COVID-19 pandemic. Interviewers were trained by the principal investigators, attained inter-rater reliability, and attended reliability meetings to guard against rater drift. The average inter-rater kappa was 0.82.

#### Structural MRI Acquisition.

Participants were scanned using a 3T Siemens TIM Trio MRI scanner (Siemens Healthineers). Sagittal T1-weighted structural images were acquired using a 32-channel receiver head coil and a 6.1-minute magnetization-prepared rapid gradient-echo sequence. This scan had the following parameters: repetition time = 1900 ms, echo time = 2.89 ms, field of view = 256 mm, voxel size = 1-mm^3^ isotropic voxels, and parallel acquisition technique mode = GRAPPA, with acceleration factor PE = 2.

#### MRI Data Processing.

High-resolution, T1-weighted MRI scans were processed using the FreeSurfer recon-all function (version 6.0) (49–51). Recon-all is a built-in FreeSurfer function that converts high-resolution 3-dimensional anatomical images into 2-dimensional inflated and pial cortical reconstructions. In addition to CT and subcortical volume, which are the default outputs of recon-all, we calculated the LGI metric, which quantifies the amount of cortex buried in sulci within specific regions of interest (ROIs) (52). The ROIs in each hemisphere of each participant were then labeled using the automatic parcellation annotation file produced in the recon-all process (53).

#### ROI Analyses.

**Figure 1.**
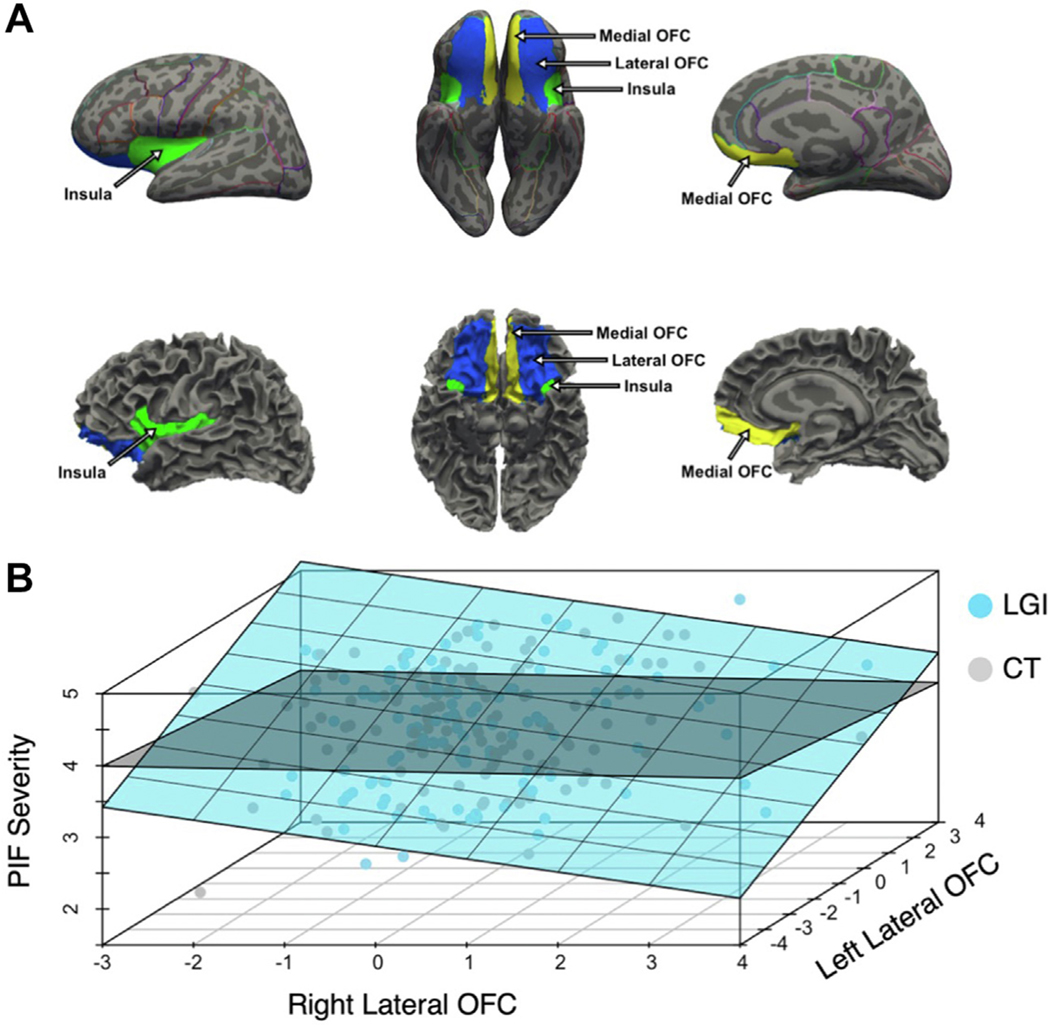
Local gyrification in the lateral orbitofrontal cortex (OFC) differentially relates to emotion-related impulsivity. **(A)** Anatomical regions were defined using the Desikan-Killiany-Tourville atlas (63). Example reconstructed surfaces shown as inflated with the atlas outlined (top) and with natural folding (bottom). Cortical regions of interest included the medial OFC (yellow), lateral OFC (blue), and insula (green). **(B)** Three-dimensional scatter plots with ordinary least squares planes of best fit. Separate models were built using the standardized local gyrification index (LGI) (blue) and cortical thickness (CT) (gray) of the right and left lateral OFC to test associations with Pervasive Influence of Feelings (PIF) severity. PIF severity correlated with low LGI in the right lateral OFC and high LGI in the left lateral OFC. PIF was unrelated to CT in the lateral OFC.

Preregistered ROIs included the OFC, insula, amygdala, and nucleus accumbens. These regions were selected because they are thought to be at the core of emotion generation [amygdala (54) and nucleus accumbens (55)] or hubs at the intersection of emotion and cognitive control [OFC (25) and insula (26)]. These targeted brain regions have each correlated, albeit inconsistently, with ERI in functional MRI studies (21) and have been implicated in psychiatric disorders (11,56–62). Using the Desikan-Killiany-Tourville atlas (63), we mapped 3 labels, the medialorbitofrontal, lateralorbitofrontal, and insula, onto each hemisphere of each participant’s cortical reconstruction (Figure 1A). The -autorecon2 stage of recon-all automatically segments more than 40 subcortical structures in each hemisphere. We used the amygdala and accumbens area labels to designate the left and right amygdala and nucleus accumbens, respectively.

For the cortical ROIs, we derived CT and LGI values. We used 2 FreeSurfer functions (*mri_annotation2label and mris_anatomicalstats*) to extract the CT of our cortical ROIs. These functions intersected the Desikan-Killiany-Tourville label files, which had been fitted to each participant’s cortical reconstruction, with their corresponding thickness files produced by recon-all. The thickness files contained measurements of the distance between the pial surface and the white matter boundary at each vertex. Each measurement was normalized based on the participant-specific thickest point in the cortex.

To calculate LGI, we included the -localGI flag within recon-all. The -localGI flag creates whole-cortex pial surface overlay files containing gyrification measurements, which reflect the degree of cortical folding. The FreeSurfer function mri_segstats then calculated the LGI statistics for the lateral and medial OFC and insula as defined by their participant-specific label files. The LGI of each region was calculated as a ratio of the amount of cortex buried in sulci to the amount visible on the surface (52).

For subcortical ROIs, we measured structural volume. The FreeSurfer function mri_segstats calculated segmented volumes (mm^3^) of the amygdala and accumbens area labels in each hemisphere of each participant’s cortical reconstruction. Univariate distributions for all brain metrics are depicted in Figures S2–S4.

We built multiple regression models to test the comparative ability of these structural brain measures to predict 2 forms of ERI (PIF and FTA), and as a comparison, LFT. We constructed 6 sets of analyses using R (64). The first 2 of these analyses were preregistered. Beyond the standard alpha of 0.05, we included Bonferroni-corrected alpha levels based on the number of predictors, which, if significant, would support our hypotheses.

In the first analysis, all CT, LGI, and subcortical volume metrics were included as predictor variables. We hypothesized that the strongest associations between brain morphology and ERI would be in the OFC. We expected that OFC morphology would not relate to LFT. We did not hypothesize whether our anatomical metrics would differentiate between FTA and PIF because of a lack of previous research. Although the phenotypes are distinct, FTA and PIF are moderately correlated and show some overlap in studies of psychopathology (10) and response inhibition (47). Therefore, we expected partial concordance in their neuroanatomical correlates. The second analysis tested the addition of quadratic metrics to the primary models. We hypothesized that quadratic (i.e., curvilinear) terms would increase model fits given that the relationship between ERI and performance on cognitive control tasks has previously been shown to be curvilinear (28,65). The third analysis tested the addition of age as a covariate to the primary models given its links to CT (66) and LGI (67). The fourth and fifth analyses isolated subsets of the linear predictor variables, LGI and CT metrics, respectively. For each model, the right and left hemisphere metrics were included. Model coefficients were interpreted using null hypothesis significance testing, and we used the multiple *R*^2^ of the models to evaluate the prediction of impulsivity scores by the collective set of neuroanatomical metrics.

The sixth analysis extended these regression analyses to test whether imbalanced LGI in the left and right lateral OFC correlated with ERI. We calculated each participant’s LGI laterality ratio in the lateral OFC as defined by Hill *et al*. (68). The calculation was as follows:

$$\frac{Right-Left}{Right+Left}$$

Positive values indicated higher LGI in the right hemisphere relative to LGI in the left hemisphere. We correlated the LGI laterality ratio in the lateral OFC with each of the 3 impulsivity factors using Pearson’s *r*.

To test the specificity of ERI results, we compared the differences in ERI and non-ERI effect size estimates using bootstrap resampling (1000 random samples with replacement). For each brain metric that was significantly associated with ERI, we estimated a 95% confidence interval from the distribution of bootstrapped effect size difference scores. We interpreted 95% confidence intervals that did not overlap with the null as evidence that brain morphology was significantly more related to ERI than non-ERI.

The preregistration document, data, and code are available at https://osf.io/tfkpb.

## RESULTS

### OFC Morphology Relates to Both Forms of ERI

Consistent with our hypothesis, the anatomical features of the OFC predicted ERI. In the full model, lower LGI, but not CT, in the right lateral OFC related to higher PIF (β = −0.315, 95% CI, −0.601 to −0.028, *t*_106_ = −2.18, *p* = .032). This effect was localized to the lateral aspect of the OFC in the right hemisphere. This effect was significant at the standard, but not at the Bonferroni-corrected, threshold. The bootstrapped 95% confidence interval comparing the strength of the effect in PIF versus LFT (i.e., non-ERI) overlapped the null (−0.612, 0.011) (Figure S5A). No other structural brain variable significantly predicted PIF, and the overall model including all variables showed weak fit (*R*^2^ = 0.11, *F*_16,106_ = 0.79, *p* = .70). For FTA and LFT, the set of cortical and subcortical predictors was nonsignificant (*p* > .05) with weak overall model fits (FTA: *R*^2^ = 0.094, *F*_16,106_ = 0.69, *p* = .80; LFT: *R*^2^ = 0.086, *F*_16,106_ = 0.62, *p* = .86) (Table S2).

Adding quadratic terms to test for curvilinear prediction did not improve model fits (Table S3). Adding age as a covariate did not alter the abovementioned findings (Table S4). Model diagnostics indicated that assumptions of residual normality and homoscedasticity were met. Because our preregistered analyses revealed a very specific effect localized to 1 cortical region (lateral OFC) and 1 morphological feature (LGI), we did not conduct preregistered feature selection procedures (i.e., lasso regression).

Consistent with previous work (39), CT and LGI were negatively correlated for 5 of the 6 hypothesized ROIs (Pearson’s *r*s from −0.15 to −0.50) (Table S5). Given the potential for the CT and LGI coefficients to be biased by collinearity when examined conjointly, we constructed separate post hoc multiple regression models for CT and LGI. Lower LGI in the right lateral OFC and higher LGI in the left lateral OFC correlated with higher PIF (Right: β = −0.248, 95% CI, −0.494 to −0.002, *t*_116_ = −2.00, *p* = .048; Left: β = 0.257, 95% CI, 0.017 to 0.497, *t*_116_ = 2.13, *p* = .035) (Figure 1B). Higher LGI in the left lateral OFC was also related to FTA severity (β = 0.254, 95% CI, 0.012 to 0.496, *t*_116_ = 2.09, *p* = .039). Although these effects were significant at α = 0.05, they did not survive Bonferroni correction. A direct comparison suggested a significantly stronger effect for PIF over non-ERI in the right lateral OFC (CI: −0.553 to −0.052), but not for PIF and FTA in the left lateral OFC (PIF CI: −0.077 to 0.510; FTA CI: −0.105 to 0.510) (Figure S5B–D). All other regressors were nonsignificant, and the models explained small proportions of the total variances in impulsivity (*R*^*2*^s < 0.07) (Table S6). None of the CT regressors significantly related to the impulsivity measures (Table S7).

Because our results were strongest for PIF and there are no prior published studies of the neuroanatomical correlates of this form of ERI, we conducted an exploratory whole-brain analysis for the purpose of generating future hypotheses. We used the FreeSurfer group-level, general linear model analysis and regressed PIF onto LGI across all cortical vertices (see Supplemental Methods and Materials). In addition to confirming the importance of the OFC, this whole-brain group analysis identified the temporal pole, lateral prefrontal cortex, frontal pole, and temporo-parietal junction as potential correlates of PIF at a less stringent (*p* < .05) vertexwise cluster detection threshold (Table S8A and Figure S6A). The temporal pole and frontal pole clusters survived a more conservative (*p* < .001) vertexwise cluster detection threshold (Table S8B and Figure S6B).

### Imbalance in Hemispheric Orbitofrontal Gyrification Correlates With PIF

**Figure 2.**
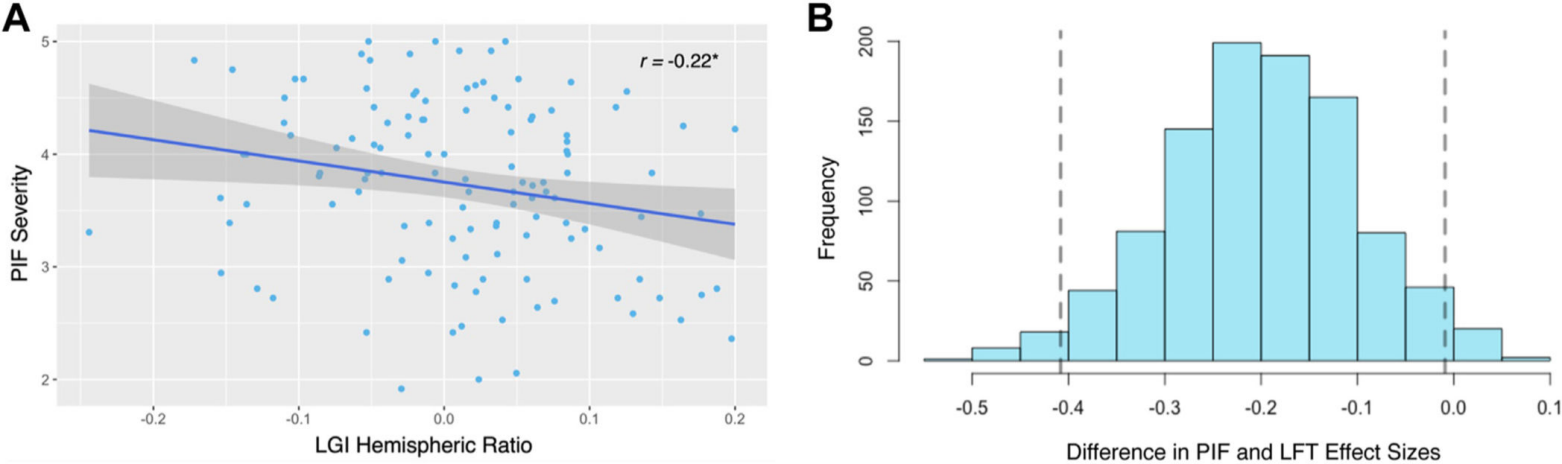
Imbalanced gyrification between the left and right lateral orbitofrontal cortex (OFC) relates to Pervasive Influence of Feelings (PIF) severity. **(A)** Participants with a low local gyrification index (LGI) hemispheric ratio in the lateral OFC—less gyrification in the right hemisphere compared with the left—were more likely on average to have higher PIF severity. Gray shading represents the 95% confidence interval around the line of best fit. *p < .05. **(B)** The correlation between the LGI hemispheric ratio and PIF was stronger than a control comparison correlation between the LGI hemispheric ratio and Lack of Follow Through (LFT). Vertical dashed lines illustrate the 95% confidence interval of the difference in the PIF and LFT effect sizes (Pearson’s *r*) from bootstrap resampling (95% CI = −0.408 to −0.009).

The opposing directionality of the lateral OFC coefficients in the right and left hemispheres fit with work by Hill *et al*. (68) who found that smaller right OFC volume relative to left OFC volume predicted higher general impulsivity scores. Using their laterality ratio calculation—positive ratios indicated higher LGI in the right lateral OFC relative to the left—we correlated LGI laterality with the 3 impulsivity factors. LGI laterality was negatively correlated with PIF scores (*r* = −0.216, 95% CI, −0.379 to −0.040, *t*_120_ = −2.42, *p* = .017) (Figure 2A) such that the lower right hemisphere LGI relative to left hemisphere LGI in the lateral OFC related significantly to PIF at standard and Bonferroni-corrected thresholds. The correlation of LGI laterality was significantly stronger for PIF than non-ERI (CI: −0.408 to −0.009) (Figure 2B). Findings for FTA showed a nonsignificant trend in the same direction as PIF (*r* = −0.153, 95% CI, −0.322 to 0.025, *t*_120_ = −1.70, *p* = .091). LGI laterality in the lateral OFC was not significantly related to LFT (*r* = −0.017, 95% CI, −0.194 to 0.162, *t*_120_ = −0.18, *p* = .856).

## DISCUSSION

Hundreds of published studies have established ERI as a robust correlate of psychopathology, including aggression (12), substance use disorders (9), depression (8), self-harm (69,70), and suicide (13), yet its relationship to brain morphometry is not well understood. To address gaps in the literature that may have contributed to past inconsistencies, we 1) used a targeted and preregistered ROI approach, 2) recruited a sizable sample (*n* = 122), 3) examined the effects within a sample that included a broad range of internalizing and externalizing syndromes, and 4) used surface-based cortical metrics, LGI and CT. To our knowledge, this study was the first to investigate ERI in relation to LGI and also the first to investigate the neuroanatomical correlates of PIF impulsivity. Together, our findings provide an important step in clarifying the neuroanatomical correlates of ERI, a foundation that can be built upon in future studies.

As hypothesized, of the brain regions we examined, the OFC was most strongly associated with ERI. In the right hemisphere, lower LGI in the lateral OFC correlated with PIF. In the left hemisphere, higher LGI in the lateral OFC correlated with both PIF and FTA severities. To our knowledge, this is the first neurobiological finding that explains some of the shared variance observed between these two ERI phenotypes. Regarding specificity, we found no significant associations between non-ERI and brain morphology in the areas we studied, and in direct comparison, local gyrification of the right lateral OFC correlated with PIF more strongly than with non-ERI.

Given the lack of prior research on PIF, we conducted an exploratory whole-brain analysis to aid future hypothesis generation. In addition to replicating the association between LGI in the lateral OFC and PIF, we identified the temporal pole, frontal pole, dorsolateral prefrontal cortex, and temporoparietal junction as candidates for future research on the neuroanatomical correlates of ERI. For example, as the right temporal pole is involved in emotion processing and regulation (71,72) and has robust connectivity with the lateral OFC via the uncinate fasciculus (73), a promising approach for future studies would be to examine how functional and anatomical features of the lateral OFC and temporal pole together contribute to ERI.

A laterality ratio, which integrated LGI in the right and left hemispheres, demonstrated that imbalanced cortical gyrification in the lateral OFC corresponded with higher PIF. The use of the laterality ratio seemed to increase the robustness and specificity of our findings in two main ways. First, the correlation of OFC laterality and PIF passed multiple comparison correction, whereas some separate OFC hemisphere coefficients were significant only at the standard alpha level. Second, LGI laterality was more strongly related to PIF than to non-ERI. This result extends the important findings of Hill *et al*. (68) with increased neuroanatomical (i.e., lateral OFC) and psychological specificity (i.e., ERI). Our results were specific to LGI and did not extend to other morphological features, such as CT. The cluster of null effects across the other ROIs in our preregistered analyses (medial OFC, insular cortex, amygdala, and nucleus accumbens) highlights the unique importance of lateral OFC gyrification for understanding ERI.

Although the OFC has been identified in only a minority of structural MRI studies of ERI (21), our findings fit with the broader literature on the OFC and human behavior beyond the studies on ERI. For example, brain lesion studies have long shown that damage to the OFC can lead to deficits in emotional functioning (74–76) and to disinhibited behavior in the context of emotion (77–79). Functional and structural MRI studies have demonstrated that the OFC is involved in value-based decision making and emotion regulation (25,80), and one recent study found that direct electrical stimulation of the lateral OFC attenuated depression symptoms (81). These studies do not directly test ERI, yet they describe its core characteristics in relation to the OFC. Our findings are also compatible with research linking inhibitory control to both ERI and the right OFC (28,70,82,83). Furthermore, the association of lower LGI in the right lateral OFC with higher ERI is consistent with previous work showing that lower LGI is associated with worse cognitive and behavioral functioning in other domains (84,85). In sum, our results dovetail with the literature on ERI, OFC function, and inhibitory control.

Beyond this conceptual convergence, our findings derive import from 1) being preregistered 2) having anatomical specificity within the OFC, 3) using surface-based cortical metrics that are sensitive to neurodevelopment, and 4) connecting to specific, differentiable, and stable impulsivity phenotypes, PIF and FTA, that have well-established and transdiagnostic relationships to psychiatric disorders. Indeed, our results in the lateral OFC appear to bridge parallel transdiagnostic literatures in psychiatry and neuroscience. While ERI has been championed as a transdiagnostic phenotype in clinical literature (18–20), the study of lateral OFC morphology and psychopathology appears to be more siloed, with few studies testing for similarities across diagnostic boundaries (62,86–89). Therefore, we hope that these findings will inform psychological frameworks that integrate the myriad studies linking lateral OFC morphology to separate psychiatric diagnoses. For example, one possible model could be that ERI is an intermediate psychological phenotype that emerges from the cortical development of the lateral OFC and leads to psychopathology. Longitudinal research will be needed to test such models. The transdiagnostic makeup of our sample may have been key to unlocking this link because neuroanatomical characteristics specific to any one diagnosis were less likely to interfere with the shared ERI signal. We hope that both the methodological approach and the empirical results from this study can support future work on ERI and generalize to other research domains that seek to integrate the transdiagnostic study of psychopathology at two crucial levels of analysis, psychiatry and neuroscience.

Beyond its strengths, this study has several limitations. Replication of these findings is needed, which we expect given the consistency of the findings with previous studies and theory. The current approach provided less clarity about FTA than it did for PIF. This may be related to the higher proportion of participants in this sample with internalizing psychopathology, which is more closely related to PIF than FTA (7). Other imaging techniques that measure the ways that brain regions dynamically respond to emotion may also be important for building the full picture. The current findings also do not provide information as to when these structural correlates of ERI arise in development. LGI, similar to ERI, is sensitive to environmental insults (38) and the development of cognitive abilities (3,40). An important next step will be to use longitudinal designs to study parallels in the cortical maturation of the lateral OFC and the emergence of ERI.

Despite their limitations, the current findings have implications for mental health treatment. Both the lateral OFC and ERI have been shown to be responsive to existing treatments, such as mindfulness (90–92), cognitive training (93–95), and cognitive behavioral therapy (96,97). These intervention studies complement the empirical link between LGI of the lateral OFC and ERI severity, and they imply that interventions targeting the lateral OFC may be promising for treating psychopathology transdiagnostically. With replication, these findings can help clinical scientists target a core feature of psychopathology at neurobiological and psychological levels of intervention, which could alleviate suffering and save lives.

## Supplementary Material

supplement

## Figures and Tables

**Table 1.** Participant Characteristics (*n* = 122)

Characteristic	*n* (%) or Mean (SD) [Range]
Gender	
Female	81 (66.4%)
Male	34 (27.9%)
Nonbinary	6 (4.9%)
Declined to respond	1 (0.8%)
Race	
Asian/Asian American	35 (28.7%)
Black/African American	8 (6.6%)
More than one race	22 (18.0%)
White/European American	51 (41.8%)
Declined to respond	6 (4.9%)
Ethnicity	
Hispanic or Latina/o	23 (18.9%)
Not Hispanic or Latina/o	99 (81.1%)
Age, Years	28.0 (8.6) [18–55]
Education, Years	15.5 (2.3) [12–21]
SCID-5 Lifetime Diagnosis	
Major depressive disorder	99 (81.1%)
Anxiety disorder	82 (67.2%)
Alcohol use disorder	27 (22.1%)
Substance use disorder	24 (19.7%)
More than one disorder	82 (67.2%)
Impulsivity Subtype	
Pervasive influence of feelings	3.73 (0.76) [1.92–5.00]
Feelings trigger action	2.86 (0.75) [1.23–4.94]
Lack of follow through	3.15 (0.80) [1.00–4.80]

SCID-5, Structured Clinical Interview for DSM-5.

**Table:** KEY RESOURCES TABLE

Resource Type	Specific Reagent or Resource	Source or Reference	Identifiers	Additional Information
Add additional rows as needed for each resource type	Include species and sex when applicable.	Include name of manufacturer, company, repository, individual, or research lab. Include PMID or DOI for references; use “this paper” if new.	Include catalog numbers, stock numbers, database IDs or accession numbers, and/or RRIDs. RRIDs are highly encouraged; search for RRIDs at https://scicrunch.org/resources.	Include any additional information or notes if necessary.
Software; Algorithm	Freesurfer	https://doi.org/10.1016/j.neuroimage.2012.01.021	RRID:SCR_001847	
Software; Algorithm	R Project for Statistical Computing	CRAN	RRID:SCR_001905	Data and R code for this study are available at: https://osf.io/tfkpb/
