# Supplementary material for: Neuroanatomical Correlates of Emotion-Related Impulsivity
Source: Biol Psychiatry. Author manuscript; Available in PMC 2023 Nov 15. (PMC9898470; doi:10.1016/j.biopsych.2022.07.018)
Supplement: supplement [file NIHMS1857465-supplement-supplement.pdf]

SUPPLEMENT

Neuroanatomical Correlates of Emotion-Related Impulsivity

Elliott *et al.*

Supplementary Methods and Materials – Whole Brain (Group) Analysis

To explore the relationship of Pervasive Influence of Feelings with whole-brain local gyrification index at the group level, we used the FreeSurfer (version 6.0) "command-line" group analysis stream. We first designed a FreeSurfer Group Descriptor File containing labels and covariates for each participant. We then created contrast matrices that specified contrast weights for Pervasive Influence of Feelings against a standard control variable.

Using the FreeSurfer (version 6.0) function "mris_preproc," each participant's data was resampled to the fsaverage template in MNI space, and each participant's data was concatenated into a single dataset. Using the FreeSurfer (version 6.0) function "mri_glmfit," the concatenated dataset was then fit to a general linear model regressing Pervasive Influence of Feelings onto local gyrification index across all cortical vertices, creating group-level contrast maps. We set two thresholds to constrain the findings - a vertex-wise, cluster detection threshold (CDT) and a clusterwise correction for multiple comparisons. We examined two CDT thresholds, $p < 0.05$ and $p < 0.001$. The clusterwise correction for multiple comparisons was set to $q < 0.05$ for all analyses.

Supplementary Figure 1. Univariate distributions of three factor impulsivity scores.

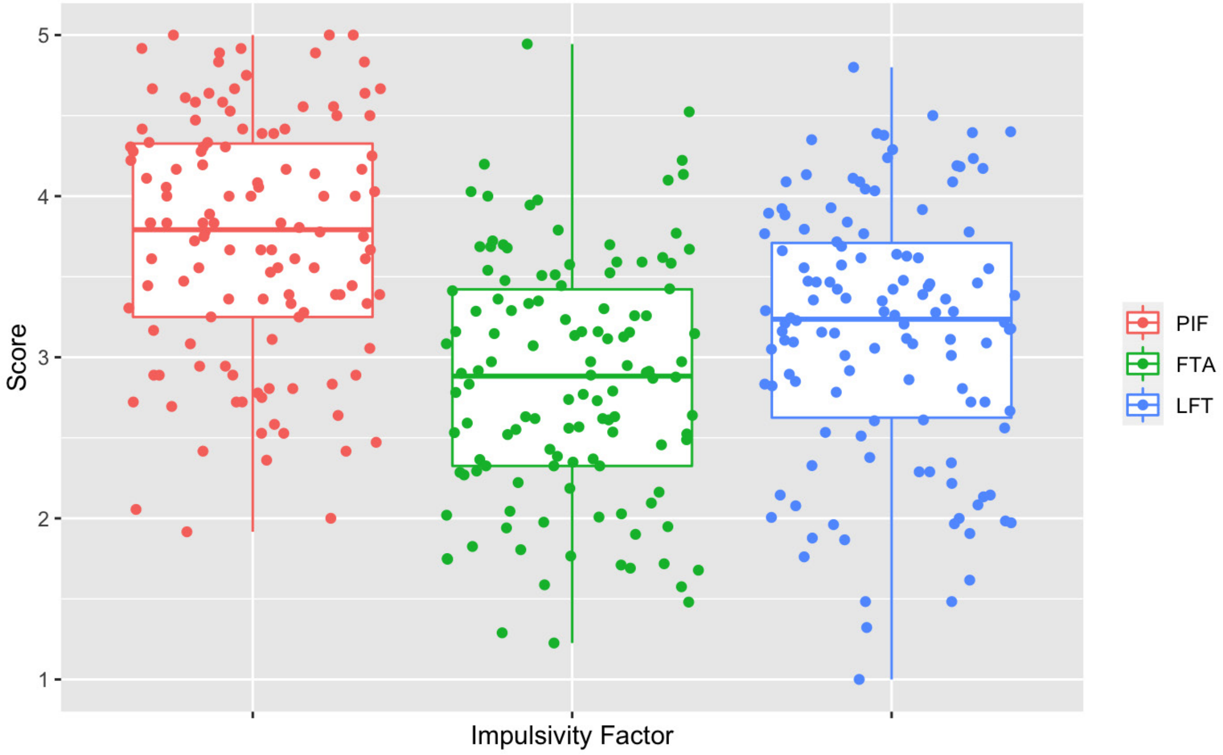

Note. FTA = Feelings Trigger Action, LFT = Lack of Follow Through, PIF = Pervasive Influence of Feelings. Box plot elements: centre line, median; box limits, upper and lower quartiles; whiskers, range of non-outlier data (1.5 times the interquartile range or the most extreme value).

Supplementary Figure 2. Univariate distributions of local gyrification index in the cortical regions of interest.

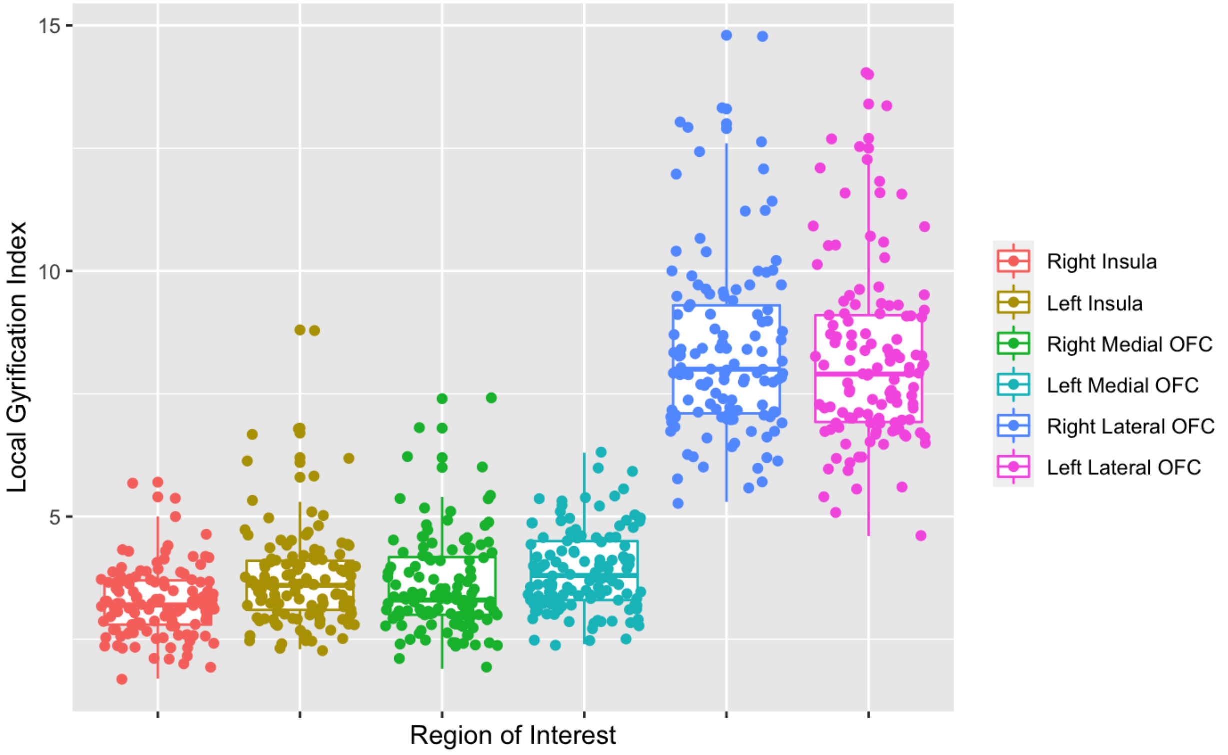

Note. OFC = Orbitofrontal cortex. Box plot elements: centre line, median; box limits, upper and lower quartiles; whiskers, range of non-outlier data (1.5 times the interquartile range or the most extreme value).

Supplementary Figure 3. Univariate distributions of cortical thickness in the cortical regions of interest.

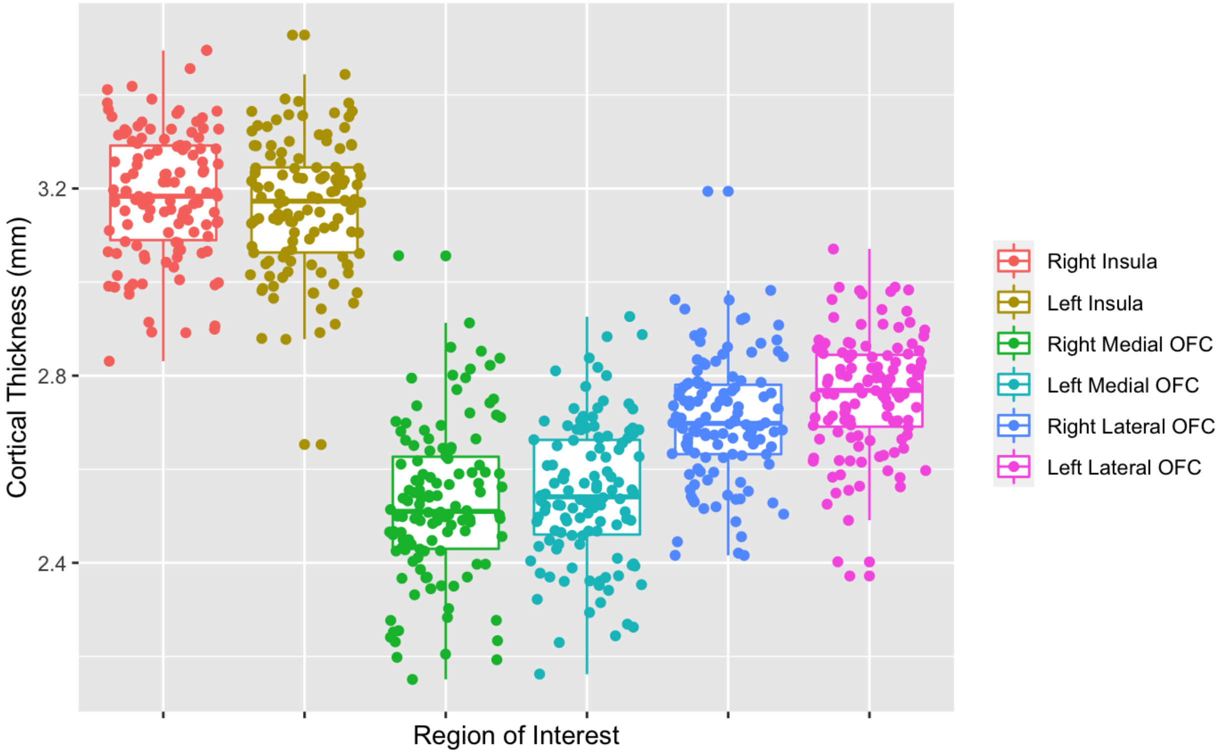

Note. OFC = Orbitofrontal cortex. Box plot elements: centre line, median; box limits, upper and lower quartiles; whiskers, range of non-outlier data (1.5 times the interquartile range or the most extreme value).

Supplementary Figure 4. Univariate distributions of structure volume in the subcortical regions of interest.

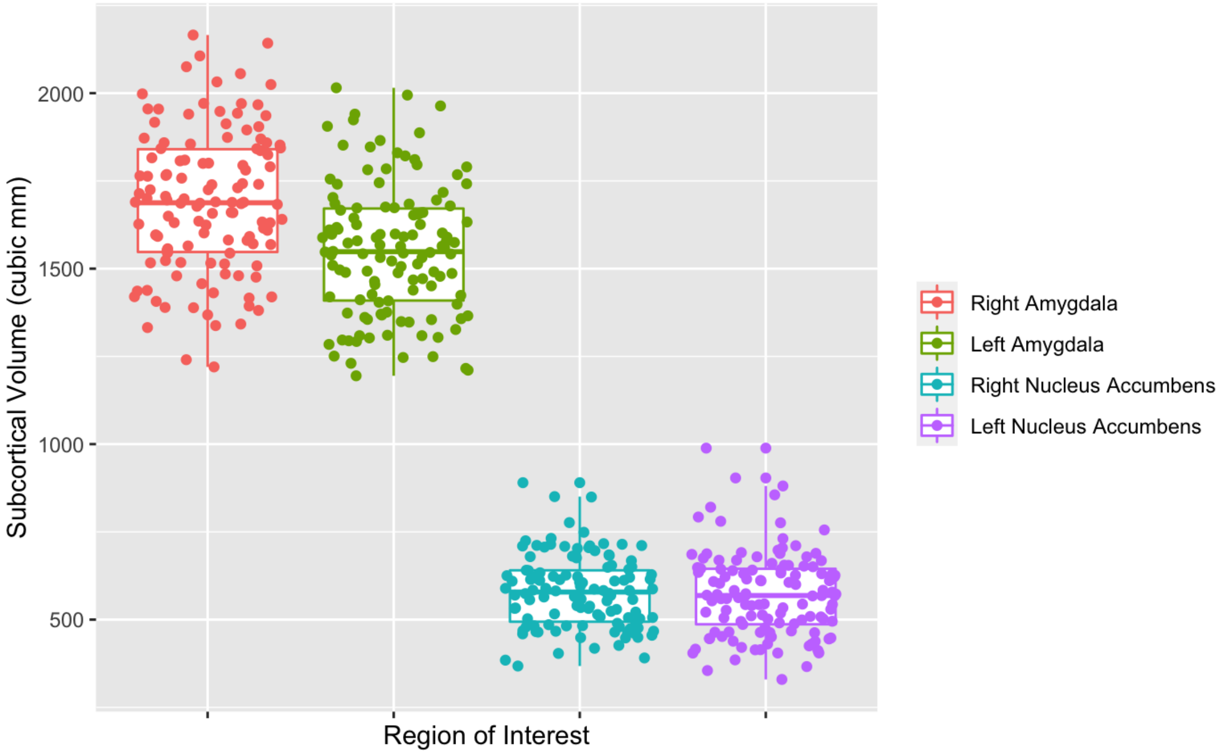

Note. Box plot elements: centre line, median; box limits, upper and lower quartiles; whiskers, range of non-outlier data (1.5 times the interquartile range or the most extreme value).

Supplementary Figure 5. Bootstrapped 95% confidence intervals comparing ERI and non-ERI effect sizes.

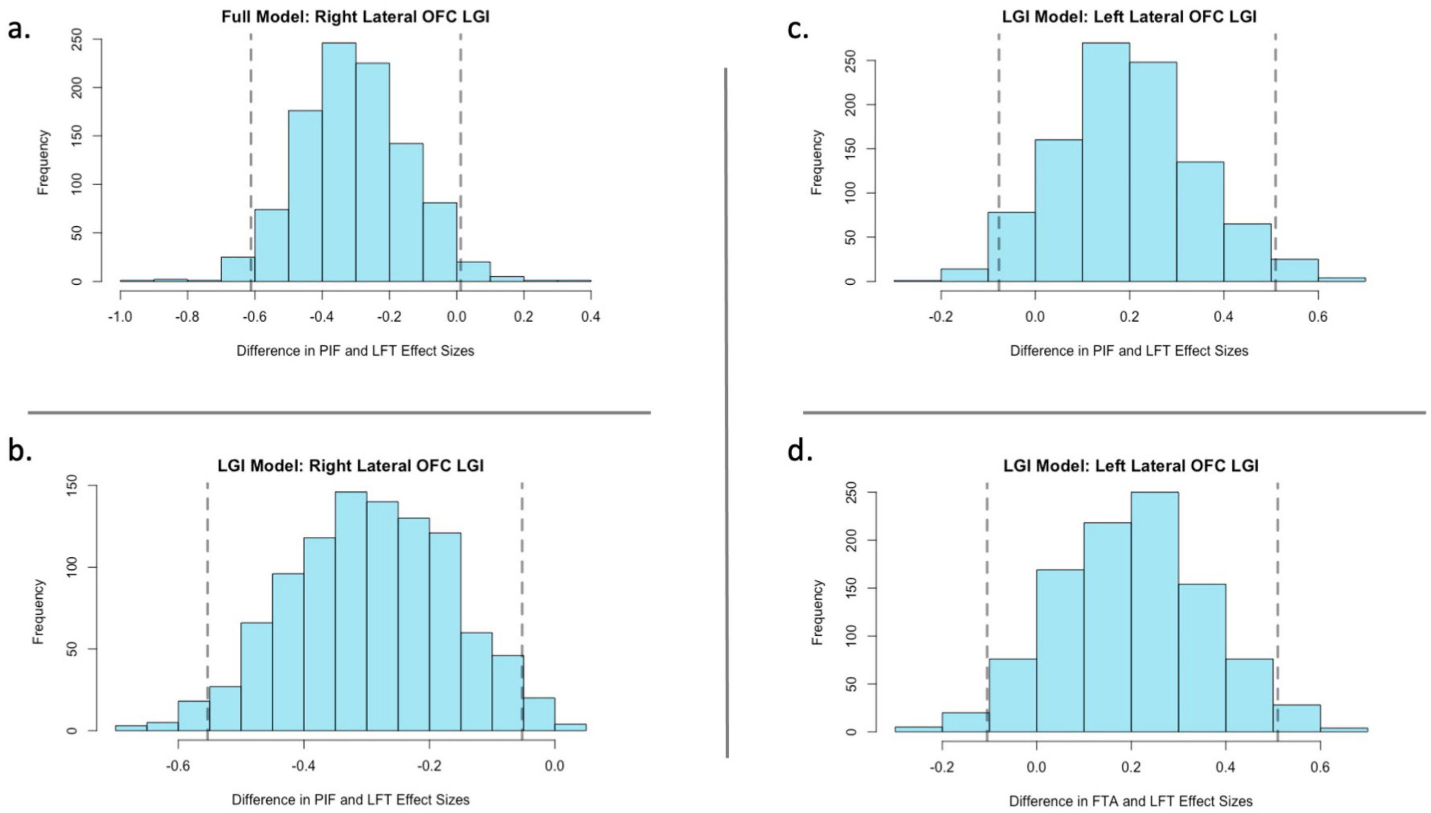

Note: Histograms estimating the effect size differences using bootstrap resampling for a. PIF vs. LFT in right lateral OFC LGI (full model), b. PIF vs. LFT in right lateral OFC LGI (LGI-only model), c. PIF vs. LFT in left lateral OFC LGI (LGI-only model), d. FTA vs. LFT in left lateral OFC LGI (LGI-only model). Vertical dashed lines represent the 95% confidence intervals.

Supplementary Figure 6. Freesurfer group-level, whole brain analyses.

a.

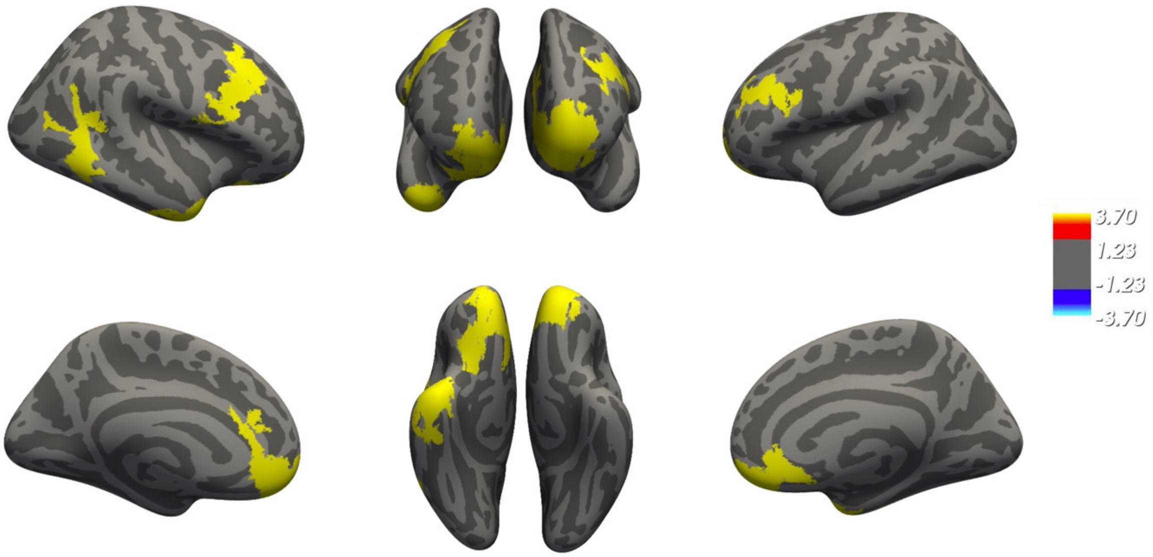

b.

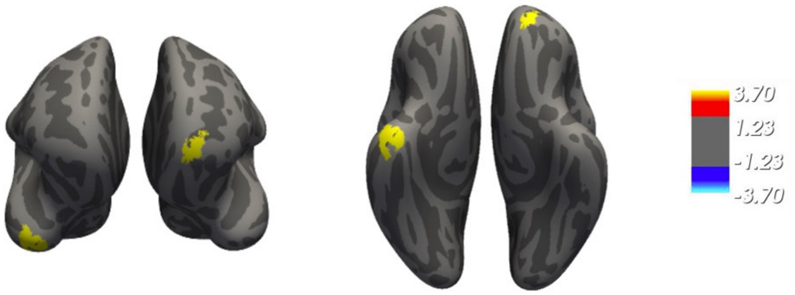

Note: Group-level general linear model regressing Pervasive Influence of Feelings severity scores onto local gyrification index across all cortical vertices at cluster-wise detection thresholds of a. $p < 0.05$, b. $p < 0.001$. Color bars denote peak statistical thresholds (t – values).

Supplementary Table 1

Means, standard deviations, and intercorrelations of Three Factor Impulsivity Index

Variable	M	SD	1	2
1. FTA	2.86	0.75		
2. PIF	3.73	0.76	.38**<br>[.22, .52]	
3. LFT	3.15	0.80	.23*<br>[.06, .39]	.34**<br>[.18, .49]

Note. M and SD are used to represent mean and standard deviation, respectively. Values in square brackets indicate the 95% confidence interval for each correlation.
* indicates $p < .05$. ** indicates $p < .01$.

Supplementary Table 2

Multivariate models regressing impulsivity on neuroanatomical metrics

Model 1: Predicting Pervasive Influence of Feelings				
Brain Metric	*β*	*S.E.*	*t*	*p*
Left Insula CT	0.04	0.14	0.30	0.762
Right Insula CT	-0.09	0.14	-0.64	0.524
Left Medial OFC CT	0.16	0.11	1.43	0.156
Right Medial OFC CT	-0.02	0.12	-0.17	0.866
Left Lateral OFC CT	-0.09	0.15	-0.62	0.534
Right Lateral OFC CT	-0.14	0.15	-0.93	0.355
Left Insula LGI	0.01	0.11	0.06	0.93
Right Insula LGI	-0.08	0.12	-0.68	0.498
Left Medial OFC LGI	0.06	0.11	0.60	0.551
Right Medial OFC LGI	0.14	0.11	1.20	0.232
Left Lateral OFC LGI	0.21	0.13	1.60	0.112
**Right Lateral OFC LGI**	**-0.31**	**0.14**	**-2.18**	**0.032**
Left Amygdala Volume	0.07	0.15	0.49	0.628
Right Amygdala Volume	-0.09	0.15	-0.61	0.543
Left Nucleus Accumbens Volume	0.04	0.15	0.30	0.762
Right Nucleus Accumbens Volume	-0.03	0.16	-0.21	0.837

Multiple R^2 = 0.106; R^2 adjusted = -0.029; F(16,106) = 0.787; *p* = 0.697

Model 2: Predicting Feelings Trigger Action				
Brain Metric	*β*	*S.E.*	*t*	*p*
Left Insula CT	-0.09	0.14	-0.66	0.514
Right Insula CT	-0.03	0.14	-0.24	0.811
Left Medial OFC CT	0.03	0.11	0.23	0.822
Right Medial OFC CT	-0.01	0.12	-0.10	0.923
Left Lateral OFC CT	0.06	0.15	0.44	0.658
Right Lateral OFC CT	-0.05	0.15	-0.34	0.735
Left Insula LGI	-0.09	0.12	-0.75	0.456

Right Insula LGI	-0.09	0.12	-0.73	0.465
Left Medial OFC LGI	-0.10	0.11	-0.97	0.333
Right Medial OFC LGI	-0.004	0.11	-0.04	0.972
Left Lateral OFC LGI	0.22	0.13	1.65	0.101
Right Lateral OFC LGI	-0.15	0.15	-1.04	0.301
Left Amygdala Volume	0.03	0.15	0.23	0.819
Right Amygdala Volume	0.13	0.15	0.91	0.367
Left Nucleus Accumbens Volume	0.14	0.15	0.91	0.365
Right Nucleus Accumbens Volume	-0.09	0.16	-0.57	0.567

Multiple R^2 = 0.094; R^2 adjusted = -0.043; F(16,106) = 0.687; p = 0.801

Model 3: Predicting Lack of Follow Through				
Brain Metric	*β*	*S.E.*	*t*	*p*
Left Insula CT	0.06	0.14	0.43	0.670
Right Insula CT	-0.19	0.14	-1.40	0.163
Left Medial OFC CT	0.02	0.11	0.22	0.830
Right Medial OFC CT	-0.08	0.12	-0.71	0.481
Left Lateral OFC CT	0.09	0.15	0.60	0.549
Right Lateral OFC CT	-0.09	0.15	-0.61	0.542
Left Insula LGI	-0.07	0.12	-0.59	0.557
Right Insula LGI	-0.17	0.12	-1.42	0.158
Left Medial OFC LGI	-0.14	0.11	-1.28	0.203
Right Medial OFC LGI	0.09	0.11	0.76	0.450
Left Lateral OFC LGI	0.04	0.14	0.32	0.746
Right Lateral OFC LGI	0.02	0.15	0.13	0.896
Left Amygdala Volume	-0.06	0.15	-0.41	0.680
Right Amygdala Volume	0.004	0.15	0.03	0.979
Left Nucleus Accumbens Volume	0.04	0.15	0.26	0.793
Right Nucleus Accumbens Volume	0.09	0.16	0.58	0.563

Multiple R^2 = 0.086; R^2 adjusted = -0.052; F(16,106) = 0.620; p = 0.861

Note. CT = cortical thickness, LGI = local gyrification index, OFC = orbitofrontal cortex, Bold = $p < 0.05$, $^{\&}$ = did not pass Bonferroni threshold α = 0.006.

Supplementary Table 3

Nested comparison of multivariate regression models with and without quadratic terms

Impulsivity Type	Model Type	RSS	*df*	*F*	*p*
Pervasive Influence of Feelings	Linear	108.15	106		
	Quadratic	95.93	90	0.717	0.770
Feelings Trigger Action	Linear	109.63	106		
	Quadratic	102.45	90	0.394	0.981
Lack of Follow Through	Linear	110.64	106		
	Quadratic	100.32	90	0.580	0.892

Note. df = degrees of freedom, FTA = Feelings Trigger Action, LFT = Lack of Follow Through, PIF = Pervasive Influence of Feelings, RSS = Residual Sum of Squares

Supplementary Table 4

Multivariate models regressing impulsivity on neuroanatomical metrics and including age

Model 1: Predicting Pervasive Influence of Feelings				
Brain Metric	*β*	*S.E.*	*t*	*p*
Left Insula CT	0.04	0.13	0.33	0.745
Right Insula CT	-0.17	0.13	-1.23	0.221
Left Medial OFC CT	0.12	0.11	1.05	0.297
Right Medial OFC CT	-0.01	0.11	-0.06	0.950
Left Lateral OFC CT	-0.11	0.14	-0.79	0.431
Right Lateral OFC CT	-0.19	0.14	-1.30	0.196
Left Insula LGI	0.04	0.11	0.33	0.746
Right Insula LGI	-0.03	0.12	-0.23	0.815
Left Medial OFC LGI	0.03	0.10	0.26	0.797
Right Medial OFC LGI	0.10	0.11	0.88	0.382
Left Lateral OFC LGI	0.18	0.13	1.42	0.159
**Right Lateral OFC LGI**	**-0.34**	**0.14**	**-2.40**	**0.018**[&]
Left Amygdala Volume	0.01	0.15	0.06	0.953
Right Amygdala Volume	-0.02	0.14	-0.17	0.868
Left Nucleus Accumbens Volume	0.04	0.14	0.30	0.767
Right Nucleus Accumbens Volume	-0.05	0.15	-0.34	0.732
**Age**	-0.30	0.11	-2.70	0.009[#]

Multiple R^2 = 0.163; R^2 adjusted = 0.028; F(17,105) = 1.206; *p* = 0.273

Model 2: Predicting Feelings Trigger Action				
Brain Metric	*β*	*S.E.*	*t*	*p*
Left Insula CT	-0.09	0.14	-0.65	0.517
Right Insula CT	-0.06	0.14	-0.45	0.654
Left Medial OFC CT	0.01	0.11	0.08	0.940
Right Medial OFC CT	-0.01	0.12	-0.06	0.955

Left Lateral OFC CT	0.06	0.15	0.39	0.699
Right Lateral OFC CT	-0.07	0.15	-0.47	0.642
Left Insula LGI	-0.08	0.12	-0.64	0.519
Right Insula LGI	-0.07	0.12	-0.56	0.579
Left Medial OFC LGI	-0.12	0.11	-1.09	0.277
Right Medial OFC LGI	-0.02	0.11	-0.16	0.871
Left Lateral OFC LGI	0.21	0.13	1.57	0.120
Right Lateral OFC LGI	-0.16	0.15	-1.10	0.274
Left Amygdala Volume	0.01	0.15	0.07	0.947
Right Amygdala Volume	0.16	0.15	1.06	0.292
Left Nucleus Accumbens Volume	0.13	0.15	0.90	0.368
Right Nucleus Accumbens Volume	-0.10	0.16	-0.62	0.536
Age	-0.11	0.12	-0.98	0.328

Multiple R^2 = 0.102; R^2 adjusted = -0.043; F(17,105) = 0.703; p = 0.793

Model 3: Predicting Lack of Follow Through				
Brain Metric	*β*	*S.E.*	*t*	*p*
Left Insula CT	0.06	0.14	0.43	0.665
Right Insula CT	-0.23	0.14	-1.65	0.103
Left Medial OFC CT	0.003	0.11	0.03	0.979
Right Medial OFC CT	-0.08	0.18	-0.66	0.513
Left Lateral OFC CT	0.08	0.15	0.53	0.597
Right Lateral OFC CT	-0.12	0.15	-0.77	0.441
Left Insula LGI	-0.05	0.12	-0.47	0.643
Right Insula LGI	-0.15	0.12	-1.19	0.235
Left Medial OFC LGI	-0.15	0.11	-1.44	0.154
Right Medial OFC LGI	0.07	0.11	0.59	0.555
Left Lateral OFC LGI	0.03	0.14	0.22	0.826
Right Lateral OFC LGI	0.01	0.15	0.05	0.957
Left Amygdala Volume	-0.09	0.15	-0.61	0.543
Right Amygdala Volume	0.04	0.15	0.24	0.814
Left Nucleus Accumbens Volume	0.04	0.15	0.26	0.798
Right Nucleus Accumbens Volume	0.08	0.16	0.52	0.604

Age	-0.14	0.12	-1.24	0.217

Multiple R^2 = 0.099; R^2 adjusted = -0.047; $F(17,105)$ = 0.677; p = 0.819

Note. CT = cortical thickness, LGI = local gyrification index, OFC = orbitofrontal cortex, Bold = $p < 0.05$, [&] = did not pass Bonferroni threshold α = 0.006, [#] = effect not hypothesized.

Supplementary Table 5

Correlations (Pearson's r) between cortical thickness and local gyrification index

Brain Region	*r*	95% C.I.	*p*
Left Insula	-0.152	[-0.321 0.026]	0.094
**Right Insula**	**-0.193**	**[-0.358 -0.015]**	**0.037**
Left Medial OFC	0.016	[-0.162 0.193]	0.860
**Right Medial OFC**	**-0.212**	**[-0.376 -0.036]**	**0.019**
**Left Lateral OFC**	**-0.315**	**[-0.466 -0.145]**	**0.000**
**Right Lateral OFC**	**-0.495**	**[-0.618 -0.348]**	**0.000**

Note. C.I. = Confidence Interval, OFC = orbitofrontal cortex. Bold = $p < 0.05$.

Supplementary Table 6

Multivariate models regressing impulsivity on local gyrification index

Model 1: Predicting Pervasive Influence of Feelings				
Brain Metric	*β*	*S.E.*	*t*	*p*
Left Insula LGI	0.03	0.11	0.31	0.757
Right Insula LGI	-0.04	0.11	-0.40	0.694
Left Medial OFC LGI	0.03	0.10	0.28	0.780
Right Medial OFC LGI	0.11	0.10	1.13	0.263
**Left Lateral OFC LGI**	**0.26**	**0.12**	**2.13**	**0.035[&]**
**Right Lateral OFC LGI**	**-0.25**	**0.12**	**-2.00**	**0.048[&]**

Multiple R^2 = 0.066; R^2 adjusted = 0.018; F(6,116) = 1.371; *p* = 0.232

Model 2: Predicting Feelings Trigger Action				
Brain Metric	*β*	*S.E.*	*t*	*p*
Left Insula LGI	-0.09	0.11	-0.83	0.409
Right Insula LGI	-0.05	0.11	-0.47	0.640
Left Medial OFC LGI	-0.11	0.10	-1.10	0.270
Right Medial OFC LGI	0.01	0.10	0.13	0.896
**Left Lateral OFC LGI**	**0.25**	**0.12**	**2.09**	**0.039[&]**
Right Lateral OFC LGI	-0.11	0.12	-0.91	0.364

Multiple R^2 = 0.052; R^2 adjusted = 0.003; F(6,116) = 1.054; *p* = 0.395

Model 3: Predicting Lack of Follow Through				
Brain Metric	*β*	*S.E.*	*t*	*p*
Left Insula LGI	-0.09	0.11	-0.87	0.385
Right Insula LGI	-0.13	0.11	-1.16	0.249
Left Medial OFC LGI	-0.10	0.10	-1.00	0.320
Right Medial OFC LGI	0.13	0.10	1.26	0.209
Left Lateral OFC LGI	0.05	0.12	0.42	0.675
Right Lateral OFC LGI	0.05	0.13	0.40	0.693

Multiple R^2 = 0.043; R^2 adjusted = -0.007; F(6,115) = 0.860; *p* = 0.527

Note. LGI = local gyrification index, OFC = orbitofrontal cortex, Bold = $p < 0.05$, [&] = did not pass Bonferroni threshold $\alpha = 0.013$.

Supplementary Table 7

Multivariate models regressing impulsivity on cortical thickness

Model 1: Predicting Pervasive Influence of Feelings				
Brain Metric	*β*	*S.E.*	*t*	*p*
Left Insula CT	0.05	0.13	0.37	0.711
Right Insula CT	-0.07	0.13	-0.54	0.590
Left Medial OFC CT	0.16	0.10	1.58	0.118
Right Medial OFC CT	-0.09	0.10	-0.91	0.367
Left Lateral OFC CT	-0.07	0.13	-0.52	0.604
Right Lateral OFC CT	-0.05	0.12	-0.37	0.715

Multiple R^2 = 0.033; R^2 adjusted = -0.017; F(6,116) = 0.654; *p* = 0.687

Model 2: Predicting Feelings Trigger Action				
Brain Metric	*β*	*S.E.*	*t*	*p*
Left Insula CT	-0.05	0.13	-0.40	0.692
Right Insula CT	-0.01	0.13	-0.09	0.932
Left Medial OFC CT	0.05	0.10	0.46	0.649
Right Medial OFC CT	-0.05	0.10	-0.46	0.644
Left Lateral OFC CT	0.03	0.13	0.24	0.811
Right Lateral OFC CT	-0.01	0.13	-0.09	0.930

Multiple R^2 = 0.005; R^2 adjusted = -0.046; F(6,116) = 0.099; *p* = 0.996

Model 3: Predicting Lack of Follow Through				
Brain Metric	*β*	*S.E.*	*t*	*p*
Left Insula CT	0.03	0.13	0.25	0.801
Right Insula CT	-0.16	0.13	-1.20	0.233
Left Medial OFC CT	0.02	0.10	0.21	0.836
Right Medial OFC CT	-0.14	0.10	-1.33	0.185
Left Lateral OFC CT	0.09	0.13	0.69	0.493
Right Lateral OFC CT	-0.02	0.12	-0.13	0.900

Multiple R^2 = 0.031; R^2 adjusted = -0.019; F(6,116) = 0.626; *p* = 0.709

Note. CT = cortical thickness, OFC = orbitofrontal cortex.

Supplementary Table 8

Group-level general linear model regressing Pervasive Influence of Feelings severity onto whole-cortex local gyrification

A. Cluster detection threshold $p < 0.05$

Structure	MNI (x)	MNI (y)	MNI (z)	Size (mm^2)	Max (Peak)	CWP
Lateral OFC (R)	20.6	26.7	-14.1	2547	3.90	0.0002
Temporal Pole (R)	46.2	5.4	-35.7	2220	5.14	0.0002
Dorsolateral PFC (R)	34.3	28.0	43.5	1918	3.15	0.0002
Middle Temporal Gyrus (R)	62.9	-48.3	-1.1	1405	2.79	0.0002
Frontal Pole (L)	-16.6	54.9	-10.3	2832	4.12	0.0002
Dorsolateral PFC (L)	-39.1	30.8	29.4	1253	3.15	0.0002

B. Cluster detection threshold $p < 0.001$

Structure	MNI (x)	MNI (y)	MNI (z)	Size (mm^2)	Max (Peak)	CWP
Temporal Pole (R)	46.2	5.4	-35.7	604	5.14	0.0002
Frontal Pole (L)	-16.6	54.9	-10.3	232	4.12	0.0014

Note: CWP = clusterwise corrected p-value, MNI = Montreal Neurological Institute
